# Autoantibodies to endostatin in patients with breast cancer: correlation to endostatin levels and clinical outcome

**DOI:** 10.1038/sj.bjc.6603037

**Published:** 2006-03-21

**Authors:** T Bachelot, D Ratel, C Menetrier-Caux, D Wion, J-Y Blay, F Berger

**Affiliations:** 1 Centre Léon Bérard, Equipe ‘Cytokines et Cancer’, INSERM U-590, 28 rue Laennec, 69008 Lyon, France; 2 Laboratoire de Neurosciences Précliniques UJFG, Centre Hospitalier Universitaire Michallon, Unité INSERM U-318, BP 217, 38043 Grenoble, France

**Keywords:** collagen XVIII, endostatin, autoantibodies

## Abstract

Circulating autoantibodies to self-antigens overexpressed by cancer cells are common in cancer patients. As specific proteins are expressed during neoangiogenesis, a similar phenomenon might occur with particular antigens of tumour vessels. Collagen XVIII, from which endostatin is cleaved, is highly expressed in the perivascular basement membrane of tumour-associated blood vessels and autoantibodies to endostatin have been reported in cancer patients. The present study analyses the incidence of naturally occurring autoantibodies to endostatin in the sera of breast cancer patients and their relation to endostatin serum levels and patient clinical outcome. Serum samples from 36 patients with localised breast cancer and 59 patients with a fully documented history of metastatic breast cancer were used. The immunoreactivity of serum samples was tested against purified recombinant human endostatin and endostatin levels were determined by immunoassay. We could detect anti-endostatin antibodies in the sera of 66% of the patients with localised disease and 42% of the patients with metastatic disease (*P*=0.03). There was no correlation between the presence of antibodies to endostatin and circulating levels of endostatin. The detection of autoantibodies to endostatin was associated with better prognosis in metastatic breast cancer patients (median survival time: 20 *vs* 8 months, *P*=0.03), as was the presence of low levels of serum endostatin (median survival time: 20 *vs* 9 months, *P*=0.007). These results show that a natural immune reaction against endostatin can occur in breast cancer patients. This could have important therapeutic implications with regard to endostatin therapy and raises the question of a possible role of this humoral reaction against endostatin in the neoplastic process.

Autoantibodies are frequently observed in the sera of patients with malignancies and their titres have been correlated to patients' survival or other clinicopathological parameters (Soussi, 2000; von Mensdorff-Pouilly *et al*, 2000). Cancer-related autoimmunity often appears to be directed against mutant forms of proteins or is associated with the overexpression of autoantigens in autologous tumour cells (Boon and Old, 1997; Soussi, 2000). As specific proteins are expressed during neoangiogenesis, a similar phenomenon might occur with particular antigens of tumour vessels. Endostatin, one of the most potent known natural inhibitors of angiogenesis (O'Reilly *et al*, 1997), is a C-terminal fragment of collagen XVIII, which is mainly localised in the basement membrane zones of the vessels (Muragaki *et al*, 1995), particularly in newly formed, tumour-associated blood vessels (St Croix *et al*, 2000). Elevated serum levels of endostatin have been found in metastatic cancer patients (Feldman *et al*, 2002) and have been correlated to the clinical course of the disease and to tumour vascularity in various tumour types (Bono *et al*, 2003). We have previously described autoantibodies to endostatin in several patients with cancer (Ratel *et al*, 2000) and have been able to show the existence of both circulating and tumour endostatin autoantibodies.

This study was undertaken to investigate the possible presence of autoantibodies to endostatin in the sera of breast cancer patients, as well as its relation to endostatin serum levels and patient clinical outcome.

## PATIENTS AND METHODS

### Patients and controls

This study includes 59 patients with metastatic breast cancer, 36 patients with localised breast cancer and 24 healthy women as control subjects. After obtaining patient informed consent, serum samples were collected, aliquoted and stored at −80°C. All serum samples were kept frozen at −80°C, then thawed shortly before use. Metastatic breast cancer patients were treated at our institution between January 1995 and November 2000, and their main characteristics are shown in Table 1. The 36 patients with localised breast cancer were treated at our institution between November 1994 and June 2003; they had stage 1 breast cancer (11 patients), stage 2 breast cancer (13 patients) or stage 3 breast cancer (12 patients). The median age was 56 years (range 33–85). In all cases, serum sampling was performed at the time of surgery. The control subjects for autoantibody prevalence were 24 healthy women, with a median age of 53 years (range 28–59). The sera of 16 additional healthy women were used as control for normal endostatin levels.

### Immunoreactivity of serum samples against recombinant human endostatin

Recombinant human endostatin was produced in yeast (*Pichia pastoris*) as a single polypeptide having a total molecular mass of 20 000 Da (France Biochem, Meudon, France). The amino-acid sequence of recombinant human endostatin was in total agreement with the expected amino-acid composition of the native human endostatin. In all, 1 μg of purified recombinant human endostatin was loaded into wells of a 10% sodium dodecyl sulfate–polyacrylamide gel, then electrophoresis was performed. The gel was blotted onto a polyvinylidene fluoride membrane (PVDF, Millipore, Saint Quentin, France). The blot was blocked in Tris-buffered saline (TBS)–10% non-fat milk. Polyvinylidene fluoride membrane strips were cut and separately reacted overnight at 4°C with each healthy donor's or patient's serum sample at a ratio of 1/100. The strips were washed three times, blocked in TBS–5% non-fat milk, and reacted with goat antibodies to human IgG conjugated to horseradish peroxidase (Sigma, Saint Quentin Fallavier, France). After washing, the blot was developed with diaminobenzidine substrate (Vector Laboratories, Burlingame, CA, USA). The positive control was human endostatin exposed to antibodies to murine endostatin (R&D System, Abingdon, UK).

### Western blot analysis of serum endostatin

A volume of 100 *μ*l of each serum sample was treated at 4°C with SwellGel Blue Albumin Removal Kit (Pierce, Rockford, IL, USA) according to the manufacturer's instructions. The filtrate was then collected, and protein content was quantified by a Bradford assay (Protein Assay; BioRad, Munich, Germany). Serum protein (100 *μ*g) was separated by sodium dodecyl sulphate–polyacrylamide gel electrophoresis (12% polyacrylamide) under reducing conditions and transferred to a PVDF membrane (Protran; Schleicher and Schuell, Dassel, Germany). The membrane was then blocked in 10% nonfat milk in TBS for at least 30 min, then incubated overnight at 4°C with rabbit anti-human endostatin polyclonal antibody (Oncogene Research Products, San Diego, CA, USA) in 3% BSA/TBS-Tween. After washing, PVDF membranes were incubated for 2 h with a horseradish peroxidase-conjugated antirabbit IgG antibody (BioRad). Signals were developed using the chemiluminescent detection kit (ECL plus Western Blotting Detection System; Amersham Pharmacia Biotech, Buckinghamshire, UK) as indicated by the supplier. Bands on microfilm (Hyperfilm, Amersham Pharmacia Biotech) were scanned.

### Determination of endostatin serum levels

Serum endostatin protein levels were determined by a competitive enzyme immunoassay in which the amount of biotinylated endostatin captured by the antibody decreased as the concentration of endostatin in the serum increased. The assay was then visualised using a streptavidin alkaline phosphatase conjugate and an ensuing chromagenic substrate reaction (Accucyte® human endostatin kit from Cytimmune Sciences Inc., College Park, MD, USA). All serum level determination experiments were performed in triplicates. The detection limit of the test was 1.95 ng ml^−1^; lower levels were considered undetectable.

### Statistical analysis

Statistical analyses were carried out according to the procedures of the SPSS® package 8.0 (Chicago, IL, USA, 1999). Patient characteristics were compared using Student's *t*-test for quantitative variables. Contingency tables were analysed using Pearson's *χ*^2^ test. Survival data, considered from the day of sampling to the time of death or the time of last follow-up, were assessed by means of the Kaplan–Meier method.

## RESULTS

### Circulating antibodies to endostatin in breast cancer patients

**Figure 1:**
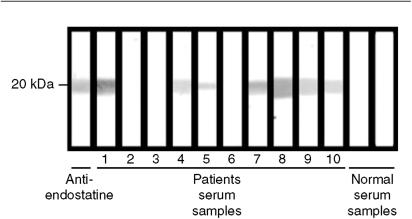
Reactivity of serum samples from patients with metastatic breast cancer, control serum samples and anti-endostatin antibodies (positive control), with recombinant human endostatin. Western immunoblot shows that serum samples from patients 1, 4, 5, 7, 8, 9, 10 and positive control (anti-endostatin antibodies) were strongly reactive to recombinant endostatin, whereas serum samples from other patients and from normal controls were negative.

The presence of endostatin autoantibodies in the serum was examined using a Western blotting approach in which immobilised recombinant human endostatin was incubated with the sera of healthy controls or of patients with metastatic breast cancer. An example of such experiments is shown in Figure 1. Antibodies to endostatin were detected in the sera of 24 out of 36 patients with localised breast cancer (66%), 25 out of 59 patients with metastatic breast cancer (42%), and 4 out of 24 healthy women (16%). Differences were statistically significant (*χ*^2^=12, *P*<0.0001 between all breast cancer patients and controls; *χ*^2^=5.2, *P*=0.03 between localised and metastatic breast cancer patients).

### Circulating endostatin in the sera of breast cancer patients

**Figure 2:**
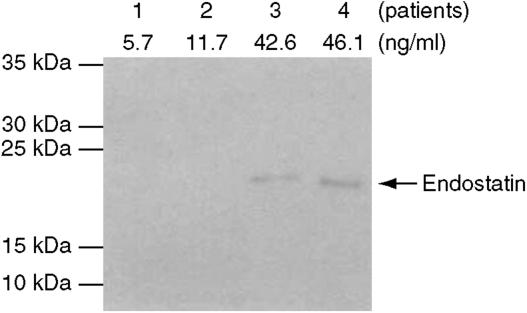
Detection of endostatin by Western blot analysis. The sera of 12 patients evaluated for endostatin concentration by ELISA were separated on polyacrylamide gel and incubated with a specific endostatin antibody as described in Materials and Methods. Results of four patients are shown. Marker bands (10, 15, 25, 30 and 35 kDa) are depicted. No specific signal could be detected in the sera with 5.7 and 11.7 ng ml^−1^ of the inhibitor.

As the appearance of autoantibodies can be attributable to a change in the status of the protein during carcinogenesis (Soussi, 2000), we searched whether serum endostatin levels were altered in metastatic breast cancer patients. Circulating serum endostatin was found detectable in most patients tested (88%), with a median of 17 ng ml^−1^ (range 0–47). By comparison, the 16 control subjects had a median serum level of endostatin of only 7 ng ml^−1^ (range 1–51), but this difference was not statistically significant (*P*=0.5). Whereas multiple C-terminal fragments of collagen XVIII have been detected in the human circulation and may not necessarily exhibit antiangiogenic activities (Sasaki *et al*, 2002), we performed additional Western blot analyses on 12 samples to identify soluble endostatin molecules. A specific endostatin band (around 21 kDa) was detected, thus corroborating the data obtained by competitive EIA (Figure 2).

### Correlation to the clinical characteristics of metastatic breast cancer patients

**Figure 3:**
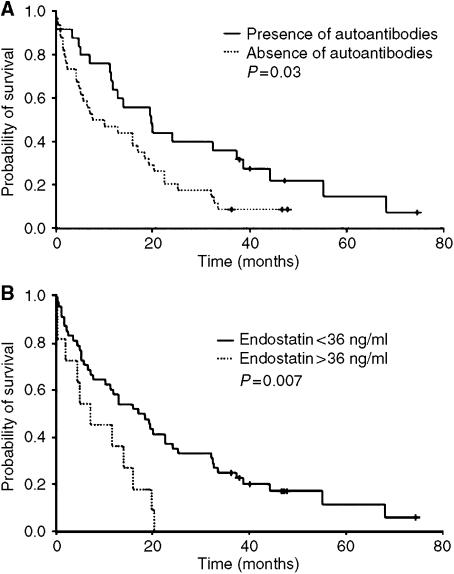
(**A**) Survival of patients as a function of the detection of autoantibodies to endostatin. Kaplan–Meier analysis showed that patients with detectable endostatin autoantibodies had significantly longer overall survival (*P*=0.03). (**B**) Survival of patients as a function of serum endostatin levels. Kaplan–Meier analysis showed that patients with lower serum endostatin levels had significantly longer overall survival (*P*=0.007).

The detection of serum autoantibodies to endostatin was correlated to better survival in metastatic breast cancer patients. The median survival time of the 25 patients with detectable serum autoantibodies to endostatin was 20 months, *vs* 7 months for the other 34 patients (*P*=0.03, Figure 3A). As different threshold values of serum endostatin, particularly 36 ng ml^−1^, have been described as being correlated to survival (Bono *et al*, 2003), we examined the prognosis of patients with high levels of serum endostatin. The 48 patients whose serum endostatin was below 36 ng ml^−1^ had a significantly better survival than the 11 patients whose endostatin level was above this value (median survival time 12 *vs* 7 months, *P*=0.007, Figure 3B). When the median serum concentration of endostatin was used as a threshold (17 ng ml^−1^), the patients within the low-level group had a median survival of 19 months, whereas the 30 patients within the high-level group had a median survival of 11 months, but this difference was not statistically significant (*P*=0.5).

As cancer-induced immunosuppression could interfere with immune response against endostatin, we assessed a possible correlation between the lymphocyte count and the detection of autoantibodies to endostatin in metastatic breast cancer patients (one missing value). Fifteen patients had normal lymphocyte count (i.e. ⩾1.5 G l^−1^), of whom 10 (66.6%) had detectable serum levels of autoantibodies to endostatin. By contrast, the serum of only 14 of 43 patients (32.5%) with a lymphocyte count below 1.5 G l^−1^ showed immunoreactivity against endostatin (*χ*^2^=5.3, *P*=0.03).

Collagen XVIII is particularly abundant in the liver, so we assessed whether patients with liver metastases had higher levels of serum endostatin or incidence of autoantibodies. This was not the case. The median serum level of endostatin in the 36 patients with liver metastases was 18 ng ml^−1^ (range 0–47) *vs* 15 ng ml^−1^ (range 0–45) in the 23 patients without liver metastasis. This difference was not significant (*P*=0.8). Serum levels of autoatibodies to endostatin were detectable in 15 of the 36 patients with liver metastases *vs* 10 of the 23 patients without liver metastasis (*χ*^2^=0.2, *P*=0.9).

With regard to treatment, this population was too heterogeneous to make any relevant correlation between endostatin serum levels or autoantibody incidence and the chemotherapy regimen received by the patient.

## DISCUSSION

Our study shows that antibodies to endostatin can be found in the sera of localised and metastatic breast cancer patients. The incidence of these autoantibodies is higher in patients with localised disease, and is associated with a better prognosis in patients with metastatic disease.

High serum endostatin levels have been described in metastatic cancer patients and most published studies show that high levels of the protein are correlated with aggressive disease or poor prognosis, which is in agreement with our results (Feldman *et al*, 2002; Bono *et al*, 2003). We have found no correlation between endostatin serum levels and the detection of antibodies to endostatin. The mean endostatin serum level was 18.2 ng ml^−1^ for the 34 patients who did not have detectable levels of antibodies to endostatin *vs* 21.5 ng ml^−1^ for the other 25 patients (*P*=0.9). Increased endostatin levels were observed in the sera of some patients who did not develop autoantibodies, indicating that additional mechanisms may be required to trigger an autoimmune response.

The fact that low endostatin serum levels and the presence of autoantibodies to endostatin are associated with a better survival is puzzling, as endostatin is supposed to act as an inhibitor of angiogenesis, tipping the balance of the angiogenic switch towards the ‘off’ position, thus inhibiting cancer progression. It has been suggested that angiogenesis-inhibiting and -promoting factors are both upregulated in aggressive diseases, angiogenesis inhibitors (like endostatin) being released as a consequence of the activity of angiogenesis promoters (like VEGF) (Feldman *et al*, 2002). Another hypothesis to explain this ‘paradox’ is that elevated serum endostatin mostly reflects ongoing angiogenesis, as it is known that (i) matrix metalloprotease, which is activated during angiogenesis, can cleave collagen and modify its structure (Xu *et al*, 2001; Hangai *et al*, 2002), and (ii) collagen XVIII is mainly synthesised in the perivascular basement membrane of tumour-associated blood vessels (Muragaki *et al*, 1995; St Croix *et al*, 2000; Guenther *et al*, 2001). This can explain that large and aggressive tumours will generate more soluble endostatin than small ones (Feldman *et al*, 2001). Finally, it should be noted that the functional status of the soluble endostatin detected in this study has not been ascertained, similar to other studies using the same competitive EIA (Feldman *et al*, 2002; Bono *et al*, 2003).

Overexpression of tumour antigens or expression of cryptic antigens following structural modification can lead to an improper immune reaction against this protein (von Mensdorff-Pouilly *et al*, 2000). Such a phenomenon might explain the presence of autoantibodies to endostatin in our patients; even so, endostatin may be expressed mostly by tumour-associated stromal cells. This hypothesis is supported by our previous work in glioma patients in which we reported an intense endostatin immunoreactivity in tumour vessels, along with immunoglobulin deposits in the same areas (Ratel *et al*, 2000).

The role of autoantibodies to tumour antigens in the neoplastic evolution is a matter of debate. Such antibodies may have an important activity in the neoplastic process. Experiments conducted on several preclinical models have shown that an immune reaction against tumour blood vessels may lead to tumour regression (Brooks *et al*, 1995). One can assume that an immune response directed against the tumour vasculature may slow down tumour progression in these patients and partially explain their better survival. This hypothesis is further supported by *in vivo* results obtained in animal models in which monoclonal antibodies to cryptic sites within collagen IV lead to inhibition of angiogenesis and tumour growth (Xu *et al*, 2001).

On the other hand, the presence of autoantibodies to endostatin may mostly reflect general auto-immunity against tumour-associated antigens (Soussi, 2000). This would explain the higher prevalence of such antibodies in localised cancer and their absence in the sera of patients with the worst prognosis, which could be related to nonspecific tumour-associated immunosuppression (Ray-Coquard *et al*, 2001; Treilleux *et al*, 2004). This hypothesis is further supported by the correlation we have shown between low lymphocyte counts and the absence of autoantibodies to endostatin.

Finally, the fact that autoantibodies to endostatin could be present in the sera of metastatic breast cancer patients may have important implications for recombinant endostatin treatments as those antibodies could interact with the recombinant protein. Such antibodies have been described in a clinical trial of recombinant human endostatin in which they were not associated with changes in endostatin pharmacokinetic (Thomas *et al*, 2003). However, the goal of this phase I clinical trial was not to assess clinical efficacy, and no response was observed. One cannot rule out a possible interaction between recombinant endostatin and pre-existing serum autoantibodies to endostatin, leading to a deterioration of its clinical efficacy.

In conclusion, this study shows that autoantibodies to endostatin can be detected in the sera of breast cancer patients and that the presence of such antibodies is associated with better survival in metastatic disease. Additional preclinical and clinical studies are warranted to understand the clinical importance of the humoral response to endostatin in breast cancer patients with regard to tumour progression and therapeutic implications.

## Figures and Tables

**Table 1:** Metastatic patient characteristics

**Characteristics**	**No. of patients**	**%**
Entered on study	59	100
		
*Age (years)*		
Median	54	
Range	26–83	
		
*PS*		
0–1	41	69.5
2–4	18	30.5
		
*Receptor status (four missing values)*		
ER positive	39	71
ER negative	16	29
		
*Adjuvant chemotherapy*		
Received	28	47.5
Not received	31	52.5
		
*Prior chemotherapy for metastatic disease*		
Received	41	69.5
Not received	18	30.5
		
*Disease-free interval (months)*		
<24	27	46
>24	32	54
		
*Metastatic site*		
Liver	36	61
Lung	27	46
One or two	34	58
Three or more	25	42
		
*Lymphocyte count (10^6^* *cells l*^−1^)		
Median	905	
Range	100–2700	
